# The Status of Metabolic Control in Patients with Diabetes Attending Primary Care Clinics in Madinah, Saudi Arabia

**DOI:** 10.3390/medicina61101856

**Published:** 2025-10-16

**Authors:** Eman Alfadhli, Amal M. Qasem Surrati, Ruqaya Saleh Masoud, Yaseera Ali Gadi, Walaa A. Alahmadi, Mohammed Khalid Turkistani

**Affiliations:** 1 Department of Medicine, College of Medicine, Taibah University, Medina 42353, Saudi Arabia; emfadhli@taibah.edu.sa (E.A.); waahmadi@taibahu.edu.sa (W.A.A.); 2 Family, Community, and Medical Education Department, College of Medicine, Taibah University, Medina 42353, Saudi Arabia; 3 Department of Family Medicine, Primary Health Care of the Ministry of Health, Madina 42316, Saudi Arabia; ruqayamasoud@gmail.com; 4 King Fahad Hospital, Madinah 42351, Saudi Arabia; ysoor.gadi@hotmail.com; 5 College of Medicine, Taibah University, Madina 42353, Saudi Arabia; m.turkistani44@gmail.com

**Keywords:** glycemic control, HbA1c, type 2 DM, Saudi Arabia

## Abstract

*Background and Objectives*: The comprehensive control of diabetes and its related comorbidities is essential to avoid diabetes complications and reduce diabetes care expenses. Nevertheless, several reports have uncovered a gap in diabetes management and confirmed suboptimal glycemic control globally. This study aims to assess metabolic control among patients with diabetes attending primary care clinics (PCCs) in Madinah, Saudi Arabia. *Materials and Methods*: This cross-sectional descriptive study took place in Madinah city, Saudi Arabia, in 15 primary care centers. A consecutive series of 692 adult diabetic patients who attended the clinics in one year were included. The primary outcome measures were achieving blood glucose, blood pressure, and lipids goals. The achievement of adequate metabolic control followed the American diabetes association (ADA) guidelines. *Results*: The majority (98%) of the patients had type 2 diabetes (T2DM) with a mean age of 55.1 ± 11.6 years and a mean diabetes duration of 11.02 ± 7.8 years. The mean HbA1c was 8.39 ± 1.7, and glycemic goals (HbA1C < 7%) were achieved in 15.7%. The achievement of LDL, triglyceride, and HDL goals were as follows; 46.4%. 53.3%, and 70.8%, respectively. In total 66.3% of subjects achieved systolic blood pressure goals, and 88.7% achieved diastolic blood pressure goals. Younger age, longer diabetes duration, and higher LDL levels were associated with poor glycemic control. *Conclusions:* Glycemic control is inadequate among patients with diabetes at PCCs in Madinah, Saudi Arabia. A patient-centered approach and individualized management plan considering all risk factors are required.

## 1. Introduction

Diabetes mellitus and its microvascular and macrovascular consequences constitute a substantial public health challenge worldwide. Despite clear evidence that controlling HbA1c, blood pressure, and LDL-C together reduces diabetes complications and mortality, studies in Asian countries have shown that achieving all three targets concurrently remains low, typically between 10% and 30% [1,2]. Consequently, the thorough management of diabetes and its associated comorbidities, including hypertension and dyslipidemia, is crucial to prevent diabetic complications and minimize diabetes care costs. Nonetheless, numerous publications have revealed deficiencies in diabetes management and validated the existence of inadequate glycemic control globally [3,4,5,6,7]. The diabetes burden in Saudi Arabia is substantial, with its prevalence rising significantly. The IDF estimates that as of 2024, 23.1% of adults aged 20–79 years are living with diabetes, placing Saudi Arabia among the top ten countries globally with the highest prevalence rates [8]. Saudi Arabia’s Ministry of Health prioritizes the enhancement of preventive and therapeutic healthcare services, with a particular focus on combating diabetes [9].The need to investigate the underlying causes of suboptimal diabetes care and to develop effective management strategies has been recognized for a long time [10]. Factors such as older age, higher body mass index (BMI), and lower educational levels were also associated with poorer glycemic control. However, identifying the most influential determinants has proven difficult [11,12].

Over the past decade, considerable progress has been achieved in the care of diabetes and its related comorbidities, integrating novel medications, technology, and comprehensive international guidelines. These enhancements are anticipated to result in improved metabolic regulation in individuals with diabetes. Although extensive data on glycemic control and the determinants of inadequate glycemia have been published from various regions in Saudi Arabia [13], there is a paucity of such information specifically pertaining to the Madinah region.

This study aimed to assess the status of glycemic, lipid, and blood pressure control among patients with diabetes in Madinah PCCs, using ADA criteria, and to identify independent predictors of poor glycemic control. This information will help guide targeted interventions and inform regional health policy.

## 2. Materials and Methods

### 2.1. Study Design and Sample Collection

We conducted a facility-based cross-sectional study across 15 primary care centers (PCCs) in the Madinah region, Saudi Arabia. The required sample size was calculated using OpenEpi (https://www.openepi.com/SampleSize/SSCohort.htm, (accessed on 6 September 2025)) for a prevalence study (assumed prevalence of poor glycemic control 50%, 99% confidence level, 5% margin of error, design effect = 1.0), yielding *n* = 664; the final enrolled sample was *n* = 692.

### 2.2. Ethical Approval

This study received ethical approval from the Institutional Review Board at the General Directorate of Health Affairs in Madinah, Saudi Arabia, IRB-165. All study procedures were conducted according to the principles of the Declaration of Helsinki.

### 2.3. Methods

Participants were consecutively and conveniently recruited from attendees of 15 PCCs during 2016–2017. Inclusion criteria comprised adult patients with diabetes aged 18 years and above who had a follow-up duration at PCC of at least one year. Exclusion criteria involved individuals with malignancies, advanced chronic diseases such as end-stage renal failure or liver failure (to avoid confounding due to severe systemic illness), those using steroids, and pregnant women.

Following an explanation of this study and obtaining written informed consent, participants underwent interviews during their routine follow-up clinic visits, and the following information was collected: age, sex, duration of diabetes, smoking status, comorbidities such as hypertension, dyslipidemia, and ischemic heart disease, as well as diabetes microvascular complications including neuropathy, nephropathy, and retinopathy.

Anthropometric measurements, including weight and height, were taken for all subjects, and blood pressure was measured. Body mass index (BMI) was calculated using the formula weight/height^2^ (kg/m^2^).

The most recent laboratory results for HbA1c, creatinine, and fasting lipid profile (total cholesterol, high-density lipoprotein (HDL), low-density lipoprotein (LDL), and triglycerides) were retrieved from the participants’ medical records. Information pertaining to documented diagnoses of hypertension, ischemic heart disease, neuropathy, nephropathy, and retinopathy was collected from the medical records as well.

In this study, hypertension was defined based on a documented diagnosis of hypertension, the use of antihypertensive medications, or having three previous high blood pressure readings (systolic ≥ 140 mm Hg or diastolic ≥ 90 mm Hg).

Dyslipidemia was defined as a documented diagnosis of dyslipidemia, the use of antidyslipidemia medications, or meeting any of the following criteria: total cholesterol > 5.0 mmol/L, LDL > 2.6 mmol/L, triglycerides > 1.7 mmol/L, or HDL < 1.0 mmol/L.

Ischemic heart disease (IHD) was defined as either a personal history of IHD or a documented diagnosis of IHD.

Microvascular complications were defined based on documented diagnoses in participants’ medical records. In addition, retinopathy was considered present if the participant had received confirmation from an ophthalmologist about the presence of retinopathy or if they had undergone laser or intravitreal eye injections. Neuropathy was defined if participants reported symptoms such as feet numbness or foot ulcers. Nephropathy was identified if the participant had an estimated glomerular filtration rate ≤ 60 mL/min/1.73 m^2^.

The achievement of adequate metabolic control was based on the ADA guidelines: HbA1C < 7%, LDL < 2.6 mmol/L, HDL > 1 mmol/L, triglyceride < 1.7 mmol/L, systolic blood pressure (SBP) < 140 mmHg, and diastolic blood pressure (DBP) < 90 mmHg [14].

### 2.4. Statistical Analysis

SPSS software (v 20.0, SPSS Inc., Chicago, IL, USA) was used to perform statistical analyses. For continuous data, the mean and standard deviation were computed, and for categorical variables, percentages were employed. The significance of differences between two continuous variables was determined using Student’s *t*-test. The chi-squared test was used to assess differences in the categorical variables. To identify factors independently associated with poor glycemic control (defined as HbA1c ≥ 7%), we conducted a multivariable logistic regression analysis. Candidate variables were selected based on clinical relevance and bivariate associations and included age, sex, BMI, duration of diabetes, LDL cholesterol, hypertension, dyslipidemia, and smoking status. Adjusted odds ratios (ORs) with 95% confidence intervals (CIs) were reported. *p* < 0.05 was the cut-off value indicating significance.

## 3. Results

A total of 692 subjects with diabetes were included: 676 patients (98%) with T2DM and 14 (2.0%) with T1DM. The mean age was 55.1 ± 11.6 years, the mean BMI was 32.1 ± 7.0 kg/m^2^, and the mean duration of diabetes was 11.02 ± 7.8 years.

Table 1a,b summarizes the baseline characteristics of the study participants and illustrates sex-related variations in glycemic control and cardiovascular comorbidities. Glycemic control did not differ significantly between males and females. However, notable differences were observed in other baseline characteristics: females had a higher BMI (33.1 vs. 29.4, *p* = 0.001) but demonstrated more favorable cardiovascular profiles, including lower triglyceride levels of 1.6 (females) vs. 1.8 (males), *p* = 0.017, higher HDL cholesterol, and a lower prevalence of hypertension, ischemic heart disease, and smoking compared to males. In contrast, the prevalence of microvascular complications such as retinopathy, nephropathy, and neuropathy did not differ significantly between sexes.

As shown in Table 2, the overall goal attainment for cardiometabolic risk factors was suboptimal. Only 15.7% of participants achieved the recommended HbA1c target of <7%. Lipid control was also limited, with less than half attaining the LDL target and just over half achieving triglyceride goals, although the majority had adequate HDL levels. Blood pressure control was comparatively better, with two-thirds meeting systolic targets and nearly 90% achieving diastolic targets.

**Figure 1:**
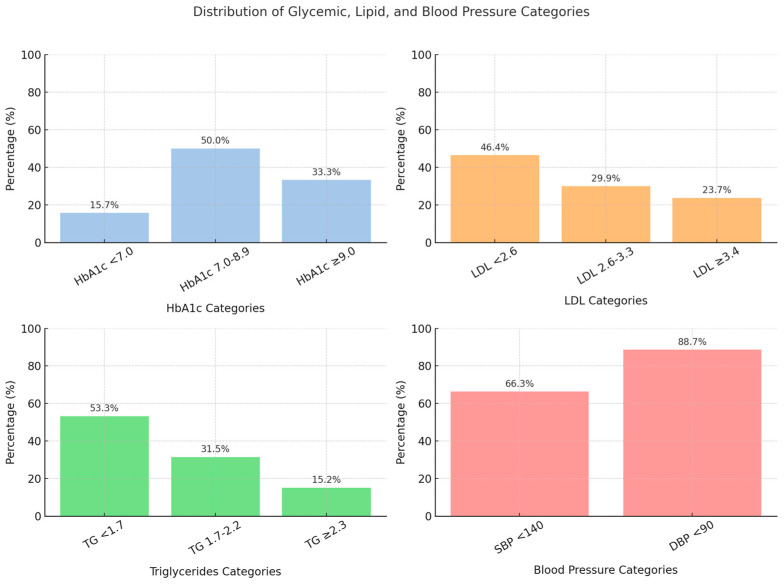
Distribution of glycemic and lipid categories among participants.

The distribution of glycemic, lipid, and blood pressure levels is illustrated in Figure 1.

Univariate analysis (Table 3) demonstrated that patients with inadequate glycemic control (HbA1c ≥ 7%) were younger and had longer diabetes duration, higher LDL, and higher fasting glucose.

Multivariable logistic regression analysis (Table 4) showed that a longer duration of diabetes, higher BMI, and elevated LDL cholesterol were independently associated with higher odds of inadequate glycemic control. Specifically, each additional year of diabetes duration was associated with a 15% increase in odds of inadequate control, and every 5 kg/m^2^ increase in BMI increased the odds by 21%. LDL cholesterol ≥ 2.6 mmol/L was also significantly associated with inadequate control (OR 1.38, 95% CI 1.10–1.75, *p* = 0.006). Conversely, older age was protective, with each 10-year increase associated with an 18% reduction in the odds of inadequate control. Other variables, including sex, hypertension, dyslipidemia, and smoking, were not independently associated with glycemic control after adjustment.

## 4. Discussion

Despite newer therapies and greater availability of diabetes technology, glycemic control remains suboptimal worldwide [1,2,3,4,5,6,7,8,9,10,11,12,13]. The current study revealed a concerning deficiency in the proportion of adult patients with diabetes attending PCCs in Madinah, Saudi Arabia, who were able to achieve glycemic goals, with only 15.7% meeting the target. This suggests that approximately only one in seven patients managed to reach the desired level of glycemic control. In a country like Saudi Arabia, where diabetes is prevalent, achieving proper control is of the utmost importance. Nevertheless, this challenge persists as glycemic control remains suboptimal. In a nationwide retrospective study conducted 2017 to 2020, it was found that 77% of patients had uncontrolled diabetes [15]. The percentage of patients with poor glycemic control was reported as 74.9% [9].

Other studies at specific healthcare facilities in Riyadh, such as the diabetes care clinics of the National Guard Health Affairs and King Khalid University Hospital’s PCC, reported diabetes control rates of 20.6% and 32.3%, respectively [10]. High rates of poor glycemic control (over 70%) and the risk factor were Risk factors included longer diabetes duration, insufficient physical activity, overweight/obesity, low adherence to medication, and lack of knowledge about HbA1c [11].

Specifically, the Diabetes Center in Madinah reported a 23.6% achievement of good glycemic control among T2DM patients [12]. In a study involving adults with T2DM attending diabetes centers in Riyadh, Hofuf, and Jeddah cities, it was found that only 24.1% of participants achieved good glycemic control [13].

The results from our study align with findings from Pakistan and Sudan studies, reflecting a global challenge in achieving optimal glycemic control among individuals with diabetes. Comparable outcomes were reported in Pakistan and Sudan, where only 16.6% and 15.0% of participants, respectively, reached the glycemic target [2,3].

In the Middle East, a specialized diabetes clinic and research center in Kuwait observed that 29.5% of patients achieved reasonable glycemic control [4]. In Jordan, glycemic control among patients with T2DM was reported to be 35% [16].

A study from Japan found that 44.9% of diabetic patients achieved glycemic control [1]. A meta-analysis, encompassing 24 studies from 20 countries, reported a pooled glycemic target achievement rate of 42.8%, with higher rates observed in North America and Europe compared to other regions [5].

One study revealed that glycemic control among type 1 diabetes patients showed no improvement between 2016 and 2018 when compared to the period between 2010 and 2012. In fact, there was a concerning worsening trend in adolescents, with only 17% meeting the HbA1c target of <7.5%, while 21% of adults met the target of <7.0% [6]. Moreover, another study from the USA unveiled that the improvements in glycemic control observed between 1998 and 2010 plateaued during 2007–2014 [7]. These findings underscore the persistent challenges and complexities associated with glycemic management, even in well-resourced healthcare systems, and emphasize the need for continuous efforts to enhance diabetes care and outcomes.

Younger ages of 13–18 yrs were observed to be associated with poor glycemic control. Previous studies have indicated that individuals under 45 years old are more likely to experience suboptimal glycemic control [17,18]. This observation could be attributed to reduced adherence to the management plan, potentially influenced by the irregularities in their lifetime routines due to active jobs and busy social events [19]. Therefore, it becomes imperative to focus on this specific demographic group in diabetes management initiatives, as they would benefit most from treatment.

The observation that a longer duration of diabetes is associated with poor glycemic control aligns with the findings of numerous previous studies [16,20]. This consistent trend highlights the notion that as the duration of diabetes increases, individuals may face greater challenges in maintaining optimal glycemic levels. The relationship between prolonged diabetes duration and poorer glycemic control emphasizes the need for ongoing monitoring, management adjustments, and tailored interventions for individuals with a longer history of diabetes to address the evolving nature of the condition over time.

The significant association between higher LDL levels and worse glycemic control observed in this study aligns with findings from numerous other studies [16,20]. This association highlights the intricate interplay between lipid metabolism and glycemic regulation and stresses the importance of addressing both aspects in the comprehensive management of diabetes.

Other factors for poor glycemic control that were out of the present study’s scope are unhealthy nutritional habits, low physical activity, low medication adherence, irregular follow-up, and psychological stresses. Drug costs can be an impediment to optimum glycemic control; however, in Saudi Arabia, visits to PCCs and prescriptions are supplied free of charge to citizens. Poor glycemic control can also be linked to the neglect of self-monitoring blood glucose, a behavior influenced by factors like needle phobia, demanding lifestyles, and the expenses associated with blood glucose strips. Earlier studies indicated that individuals with a greater understanding of diabetes tend to exhibit superior glycemic control compared to those with limited knowledge [21,22]. Knowing the HbA1c level and understanding the individual glycemic target is connected with improved glycemic control [21,22]. In a study from Saudi Arabia, it was uncomfortably observed that a third of the participants lacked awareness of their HbA1c levels, 32.0% had never heard of HbA1c, and 36.1% were unaware of their HbA1c goal [13]. Physicians and diabetes educators should convey to diabetic patients their HbA1c level at each clinic visit and the target they should achieve to improve their glycemic control.

Primary care physicians’ knowledge and the application of updated guidelines for the management of diabetes may not be optimal and may add to the hurdle to achieving glycemic control. Clinical inertia is a crucial barrier to achieving euglycemia. Therapy must be intensified whenever glycemic control deteriorates, and referral to a diabetes specialist or an endocrinologist should be performed when glycemic control is deemed complicated. Therapeutic inertia not only affects diabetes management but also affects other cardiovascular diseases management such as hypertension and dyslipidemia. Strategic plans to prevail over clinical inertia must include actions that target patients, physicians, and healthcare systems. Multifactorial interventions that act on different therapeutic goals beyond glycemia are needed [23].

In the present study, the control of LDL cholesterol was better than glycemic control, as nearly half of the patients achieved the goal. This result is comparable to the metanalysis mentioned above [5] and better than the study from Japan [1], in which only 27.1% achieved the target. The achievement of the triglyceride goal was slightly better than the goal for LDL (53.3% vs. 46.4%, respectively), comparable to the results from a study from Saudi Arabia [10], but less than the results from a metanalysis in which the pooled target achievement was 61.9% (55.2–68.2%) [5]. HDL-C was the best lipid parameter controlled in the current study as 70.8% achieved the target. This result is better than the metanalysis results in which 58.2% (51.7–64.4%) reached the goal for HDL-C [5].

Blood pressure emerged as the most effectively managed risk factor for atherosclerotic cardiovascular diseases among our participants. Approximately two-thirds of the participants achieved control over SBP, while nearly 90% attained control over DBP. This result is comparable to studies from Japan [1] and the USA [5] and better than results for other parts of Saudi Arabia. In the meta-analysis mentioned above, only 29.0% (22.9–35.9%) achieved blood pressure targets, with a greater percentage of people accomplishing the targets in North America than in the rest of the world [5].

Smoking represents a significant risk factor for cardiovascular diseases, especially in individuals with diabetes. In this study, one-quarter of the male participants were smokers, while smoking was infrequent among females. Recognizing the heightened cardiovascular risks associated with smoking in the context of diabetes, it becomes crucial to encourage and support individuals with diabetes to quit smoking.

In our patient group, glycemic control was less successful compared to the better management of other cardiovascular risk factors such as cholesterol and blood pressure. This finding is consistent with the results of the Steno-2 trial [23].

In this trial, treatment objectives for dyslipidemia and hypertension were relatively attainable. However, reaching the HbA1c goal posed the greatest challenge, with only 15% of patients in the intensive treatment group achieving the desired glycemic target [23]. Reassuringly, evidence indicates that effectively managing dyslipidemia and hypertension in patients with T2DM results in more pronounced reductions in cardiovascular events compared to exclusively prioritizing hyperglycemia control [24,25]. The Steno-2 trial and its follow-up study have compellingly demonstrated that a multidisciplinary intervention targeting hyperglycemia, hypertension, and dyslipidemia is pivotal in reducing the risk of both micro- and macrovascular complications in patients with diabetes [23,24].

This study has some limitations that should be considered. Cross-sectional studies lack temporality, making it inappropriate to assume cause and effect. Additionally, this study did not investigate factors influencing glycemic control, including lifestyle factors such as nutritional habits and physical activity, medication adherence, education level, and psychological status. While the results are pertinent to a specific region in Saudi Arabia, they nonetheless yield similar findings to those observed in other parts of the country. Recruitment was consecutive and facility-based; therefore, the findings are most representative of PCC attendees and may not fully generalize to the broader community population with diabetes. Despite these limitations, this study brings attention to the significant burden of inadequate glycemic control among patients with diabetes in Saudi Arabia. This emphasizes the imperative for effective strategies to manage diabetes, particularly within PCCs in the country.

## 5. Conclusions

The present study reveals inadequate glycemic control among patients with diabetes attending PCCs in Madinah, Saudi Arabia. Effective and ongoing education that enhances patients’ understanding of diabetes, encourages behavioral changes, and promotes a healthy lifestyle is crucial for successful diabetes management. The approach to diabetes care should be patient-centered, incorporating individualized management plans that consider all relevant risk factors. Employing a stepwise, target-driven strategy for achieving goals related to blood glucose, blood pressure, and lipid levels, including LDL and triglycerides, is essential. We recommend a multidisciplinary team approach to diabetes management, involving collaboration among physicians, diabetes educators, and clinical dietitians to ensure comprehensive and holistic patient care. Implementing smoking cessation approaches is vital for improving cardiovascular health outcomes in this population.

Continuous medical education for primary healthcare physicians is recommended to keep them updated on the latest guidelines for diabetes management. Furthermore, there is a need for ongoing research to explore additional approaches that can improve glycemic control in the context of Saudi Arabia, contributing to the development of more effective strategies for diabetes care in the region.

## Figures and Tables

**Table 1:** (**a**) Baseline characteristics of the study population (*n* = 692) stratified by gender. (**b**) Prevalence of diabetes complications and cardiovascular comorbidities stratified by sex.

Variable	Total (*n* = 692) (Mean ± SD)	Males (*n* = 183) (Mean ± SD)	Females (*n* = 509) (Mean ± SD)	*p*-Value
Age (years)	55.1 ± 11.6	56.3 ± 12.9	54.7 ± 11.1	0.270
Weight (kg)	79.4 ± 17.6	80.9 ± 17.5	78.9 ± 17.7	0.207
BMI (kg/m^2^)	32.1 ± 7.0	29.4 ± 6.0	33.1 ± 7.1	0.001
Duration of diabetes (years)	11.0 ± 7.8	12.7 ± 7.9	10.4 ± 7.8	0.002
HbA1c (%)	8.3 ± 1.7	8.5 ± 1.8	8.4 ± 1.9	0.505
Fasting glucose (mmol/L)	9.7 ± 4.2	10.0 ± 4.7	9.6 ± 4.0	0.314
LDL (mmol/L)	2.8 ± 1.04	2.8 ± 1.0	2.9 ± 1.0	0.319
HDL (mmol/L)	1.1 ± 0.4	1.1 ± 0.4	1.8 ± 0.4	0.018
Triglyceride (mmol/L)	1.7 ± 0.9	1.8 ± 1.2	1.6 ± 0.8	0.017
SBP (mm Hg)	132.3 ± 20.3	131.1 ± 21.6	132.7 ± 19.8	0.372
DBP (mm Hg)	74.3 ± 11.6	78.1 ± 11.7	73.1 ± 12.0	<0.001
**Variable**	**Total (%)**	**Males (%)**	**Females (%)**	***p*** **-value**
Diabetic retinopathy	17.9	19.1	17.5	0.590
Diabetic nephropathy	8.5	8.7	8.4	0.890
Diabetic neuropathy	23.3	25.1	22.6	0.480
Hypertension	61.2	83.9	55.1	0.001
Dyslipidemia	44.5	41.0	45.8	0.280
Ischemic heart disease	10.4	13.1	9.4	0.004
Smoking	6.8	24.0	0.6	0.001

BMI: body mass index; LDL: low-density lipoprotein; HDL: high-density lipoprotein; SBP: systolic blood pressure; DBP: diastolic blood pressure.

**Table 2:** Achievement of glycemic, lipid, and blood pressure control in 692 subjects with diabetes.

Parameters	Percentages (%)
HbA1C < 7%	15.7
LDL < 2.6 mmol/L	46.4
Triglyceride < 1.7 mmol/L	53.3
HDL >1 mmol/L	70.8
SBP <140 mmHg	66.3
DBP <90 mmHg	88.7

LDL: low-density lipoprotein; HDL: high-density lipoprotein; SBP: systolic blood pressure; DBP: diastolic blood pressure.

**Table 3:** Comparison of clinical and biochemical characteristics between participants with adequate (HbA1c < 7%) and poor (HbA1c ≥ 7%) glycemic control.

Variable	Patients with A1c < 7	Patients with A1c ≥ 7	*p* Value
Mean ± SD			
Age (years)	57.2 ± 12.4	54.6 ± 11.7	0.037
Duration of diabetes (years)	8.3 ± 7.4	11.3 ± 7.7	0.000
Weight	80.5 ± 18.2	79.2 ± 17.5	0.501
BMI	32.4 ± 6.8	32.0 ± 7.0	0.599
Height	157.5 ± 9.4	157.5 ± 9.0	0.969
Systolic BP	131.1 ± 20.8	132.2 ± 20.1	0.621
Diastolic BP	74.0 ± 11.9	74.2 ± 12.0	0.843
Fasting glucose	8.0 ± 3.7	10.0 ± 4.2	0.000
HbA1C	6.2 ± 0.5	8.8 ± 1.7	0.000
Total cholesterol	4.7 ± 1.1	4.8 ± 1.1	0.617
LDL	2.6 ± 1.0	2.9 ± 1.0	0.045
Triglyceride	1.6 ± 0.6	1.7 ± 1.0	0.176
HDL	1.1 ± 0.3	1.1 ± 0.4	0.764
Serum creatinine	77.6 ± 42	75.1 ± 42	0.521
Percentages (%)			
Sex: male/female	18.1/16.3	81.9/83.7	0.633
LDL < 2.6 mmol/L	28.7	18.5	0.025
HDL > 1 mmol/L	66.7	69.9	0.292
Triglyceride < 1.7 mmol/L	64.8	59.6	0.181
SBP < 140 mmHg	71.3	68.4	0.315
DBP < 90 mmHg	89.8	89.5	1.00

SBP: systolic blood pressure; DBP: diastolic blood pressure; LDL: low-density lipoprotein; HDL: high-density lipoprotein.

**Table 4:** Independent predictors of inadequate glycemic control (HbA1c ≥ 7%).

Variable	Adjusted OR (95% CI)	*p*-Value
Age (per 10 years ↑)	0.82 (0.70–0.96)	0.012
Diabetes duration (years)	1.15 (1.05–1.26)	0.003
BMI (per 5 kg/m^2^ ↑)	1.21 (1.02–1.43)	0.027
LDL ≥ 2.6 mmol/L	1.38 (1.10–1.75)	0.006
Male sex	1.08 (0.79–1.47)	0.624
Hypertension	0.92 (0.69–1.22)	0.580
Dyslipidemia	1.17 (0.89–1.54)	0.260
Smoking	1.41 (0.89–2.25)	0.136

## Data Availability

The raw data supporting the conclusions of this article will be made available by the authors on request.

## Abbreviations

DM	Diabetes mellitus
PCCs	Primary care centers
HDL	High-density lipoprotein
LDL	Low-density lipoprotein
BMI	Body mass index
